# Association of individual‐socioeconomic variation in quality‐of‐primary care with area‐level service organisation: A multilevel analysis using linked data

**DOI:** 10.1111/jep.13834

**Published:** 2023-03-09

**Authors:** Danielle C. Butler, Sarah Larkins, Rosemary J Korda

**Affiliations:** ^1^ National Centre for Epidemiology and Population Health The Australian National University Canberra Australia; ^2^ College of Medicine and Dentistry James Cook University Townsville Australia

**Keywords:** equity, multilevel analysis, primary care, socioeconomic inequalities, variation in care

## Abstract

**Rationale, Aims and Objectives:**

Ensuring equitable access to primary care (PC) contributes to reducing differences in health related to people's socioeconomic circumstances. However, there is limited data on system‐level factors associated with equitable access to high‐quality PC. We examine whether individual‐level socioeconomic variation in general practitioner (GP) quality‐of‐care varies by area‐level organisation of PC services.

**Methods:**

Baseline data (2006–2009) from the Sax Institute's 45 and Up Study, involving 267,153 adults in New South Wales, Australia, were linked to Medicare Benefits Schedule claims and death data (to December 2012). Small area‐level measures of PC service organisation were GPs per capita, bulk‐billing (i.e., no copayment) rates, out‐of‐pocket costs (OPCs), rates of after‐hours and chronic disease care planning/coordination services. Using multilevel logistic regression with cross‐level interaction terms we quantified the relationship between area‐level PC service characteristics and individual‐level socioeconomic variation in need‐adjusted quality‐of‐care (continuity‐of‐care, long‐consultations, and care planning), separately by remoteness.

**Results:**

In major cities, more bulk‐billing and chronic disease services and fewer OPCs within areas were associated with an increased odds of continuity‐of‐care—more so among people of high‐ than low education (e.g., bulk‐billing interaction with university vs. no school certificate 1.006 [1.000, 1.011]). While more bulk‐billing, after‐hours services and fewer OPCs were associated with long consultations and care planning across all education levels, in regional locations alone, more after‐hours services were associated with larger increases in the odds of long consultations among people with low‐ than high education (0.970 [0.951, 0.989]). Area GP availability was not associated with outcomes.

**Conclusions:**

In major cities, PC initiatives at the local level, such as bulk‐billing and after‐hours access, were not associated with a relative benefit for low‐ compared with high‐education individuals. In regional locations, policies supporting after‐hours access may improve access to long consultations, more so for people with low‐ compared with high‐education.

**Abbreviations**
AUD: Australian dollars
CD: chronic disease
CI: confidence interval
FTE: full‐time equivalent
GP: general practitioner
MBS: Medicare Benefits Schedule
NSW: New South Wales
N: number
OPC: out‐of‐pocket costs; % percentage
OR: odds ratio
PC: primary care
URP: usual resident population

## 1 INTRODUCTION

Equitable access to high‐quality primary care (PC) is expected to reduce socioeconomic inequalities in health.^1^, ^2^, ^3^ Monitoring health system performance, including PC, has historically focused on efficiency and overall effectiveness.^4^, ^5^, ^6^ More recently there has been increasing emphasis^5^, ^7^, ^8^ on performance indicators that measure equity in healthcare. However, beyond knowing whether care (and quality of care) is equitable, there is a need to understand the system‐level factors that support equity.

Service organisation and delivery characteristics of PC systems include those relevant to health services generally (e.g., availability, affordability, acceptability and accommodation) and specific to high‐quality PC (e.g., comprehensiveness, continuity and coordination).^9^, ^10^, ^11^ These characteristics of service organisation and delivery can be changed through policy and practice, implemented at either the practice level or small area level (such as neighbourhood level or local jurisdictional region). In Australia, general practitioners (GPs) are the main PC providers and the first point of contact with the health system. Like many countries with universal health insurance, Australian PC service organisation and delivery varies across small areas.^12^, ^13^, ^14^ There is also evidence that service delivery characteristics, such as supply of PC providers, scope of practice and after‐hours arrangements, are associated with PC service use^15^, ^16^, ^17^ and perceived quality‐of‐care.^18^ However, these findings were based on aggregated area‐level data or examined the association of service organisation at the practice level with individual outcomes. Further, while it is known that individuals of low socioeconomic position (SEP) use similar or more PC services for a given level of need relative to high‐SEP individuals,^19^, ^20^, ^21^ no studies to date have examined whether there are specific aspects of PC service organisation within areas that are associated with equitable quality‐of‐care.

This study examined the extent to which the organisation and delivery of PC services within areas modify individual‐level socioeconomic variation in GP quality of care. We do this with the aim of informing policies addressing inequalities in access to high‐quality care.

## 2 METHODS

### 2.1 Theoretical underpinnings and conceptual model

Conceptual frameworks propose that the health system, particularly PC, has a role in mitigating upstream determinants of health inequities, through equitable access to care—that is, equal care for equal need.^3^ Evidence also shows that where people live—physically, socially, culturally and politically—influences their health and the healthcare they receive.^22^ Part of this shared context is the local structure and organisation of PC services which, through similar mechanisms, may modify socioeconomic variation in the quality‐of‐care individuals receive, and in doing so, contribute to health equity.

Watson and colleagues' logic model^23^ outlines a pathway by which the care received may contribute to health equity. Inputs and activities of the PC system result in products and services received by individuals (measured as the frequency of use, types and qualities of care provided) that, through direct outcomes (e.g., reduced hospitalisations, stable chronic disease [CD]) and indirect outcomes (efficiency, appropriateness), result in an improved level and distribution of population health and wellness. The authors argue that it is only reasonable to measure performance based on factors for which PC organisations and providers can be reasonably held accountable for, namely the actual services delivered and qualities of these services.

In terms of the structure and organisation of PC systems, conceptual models characterise different levels of operation.^1^, ^9^, ^10^ The structural level relates to national policies (e.g., on universal access or workforce). As described in the introduction, the service delivery level relates to organisational characteristics of health services more generally and those considered core functions of high‐quality PC systems.^9^, ^10^, ^11^, ^24^ Many aspects of the service delivery level can be viewed to operate at the small area level—that is, in the more immediate vicinity of where people live rather than at a national or larger regional scale. The specific geographical scale of interest for this study is an area than can be reasonably thought to be representative of a neighbourhood or community sharing similar characteristics in terms of health services available (hereafter referred to as ‘area‐level’). While policies relating to health system delivery may be implemented at a national level, their impact on reducing unwarranted socioeconomic variation in health and healthcare occurs at the area‐ and individual‐level.^3^

Continuity of care is also viewed as a core function of PC systems.^1^, ^9^, ^11^ However, while information or management continuity may reflect how services organised (either at the area or practice level), relational continuity is experienced by the individual.^25^ Similarly, acceptability (a feature of access) primarily operates at the individual level. For our analysis, we propose that high‐quality PC, as received or experienced by individuals, is the outcome of well‐organised and managed services and effective service delivery.

**Figure 1:**
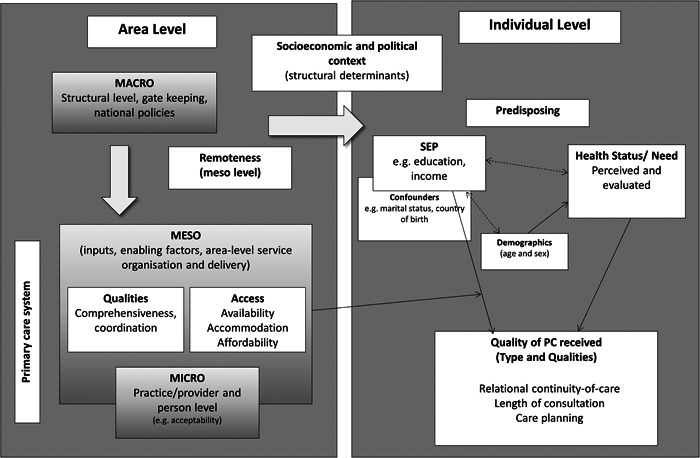
Conceptual model for examining the association of modifiable area‐level PC organisation and service delivery characteristics with socioeconomic variation in the quality of PC received. Predisposing individual‐level factors include those known to be related to use of health services.^20^, ^26^ Several Meso‐level factors relating to PC service organisation and delivery also operate in part at the practice‐ and/or provider‐level. PC, primary care; SEP, socioeconomic position.

Figure 1 illustrates the conceptual model used for this study to examine the association of *area‐level* PC service organisation and delivery with the socioeconomic variation the quality of care received by *individuals*. In this figure, factors determining use of PC services are represented as those operating at the area level and those that act at the individual level. The socioeconomic and political context straddles these levels as it is understood that, while this operates within society, social stratification manifests at the individual level (as measured by individual SEP). Further detail is given with respect to the levels of organisation within the PC system. For this analysis, we propose that inputs and enabling factors—specifically, the organisation and delivery of services within areas—may shape the relationship between an individual's SEP and the quality of care they receive.

Table 1 outlines the conceptual levels examined in this study, the related constructs of interest and the variables used to measure the relevant construct. Specific details are provided in the following text.

**Table 1:** Variables included in analysis: Level of analysis, conceptual construct and how measured in data.

Level	Conceptual construct	Proxy measure
*Area‐level*		
Explanatory variables (PC service organisation and delivery characteristics)	Access: availability (the supply of primary care providers for the population), affordability (financial barriers to receiving services) and accommodation (organisation of services to accept clients, such as hours of operations, mode of service provision [face‐to‐face or telehealth])^9^, ^24^	Per capita full‐time equivalent GPs; average OPC per service; % MBS GP services with no copayment (bulk billed); % MBS GP services after‐hours
	Comprehensiveness: services organised to provide care for most needs (chronic disease, acute, preventative, maternal health)^9^	% Chronic disease care planning and coordination MBS service per 100 long‐term conditions; Preventative health assessment MBS services per 100 eligible population
	Coordination: ability of primary care providers to coordinate use of other services (specialist and allied health), skill mix and diversity within the service, and team‐based care^9^, ^10^, ^11^	% Chronic disease care planning and coordination MBS service per 100 long‐term conditions
*Individual‐level*		
Explanatory variables	Socioeconomic position (main explanatory variable); long‐term conditions, self‐rated health, physical functioning (evaluated and perceived need); marital status, country of birth, age, sex (confounders)	See Table 2
Outcome variables (quality of PC received)		
Qualities of care	Relational continuity, interpersonal communication, respectfulness, comprehensiveness^23^, ^25^	Usual provider index, receipt of a long/prolonged MBS GP service; receipt of a chronic disease and care planning MBS service
Types of care	Chronic disease, preventative, health promotion^23^	Receipt of a chronic disease and care planning MBS service

Abbreviations: GP, general practitioner; MBS, Medicare Benefits Schedule; OPC, out‐of‐pocket cost; PC, primary care.

### 2.2 Study population and setting

The Sax Institute's 45 and Up Study is a large prospective cohort study involving 267,153 people aged 45 years and older residing in New South Wales (NSW), Australia's most populous state.^27^ Participants were randomly sampled from the Services Australia Medicare enrolment database, with over‐sampling by a factor of two of individuals aged 80 years and over and people resident in rural areas. Participants enroled in the study by completing a baseline questionnaire, distributed between January 2006 and December 2009, and providing consent for 5‐yearly questionnaires and linkage to routinely collected health data. Approximately 11% of the total NSW population aged 45 years and older was included in the study, with a response rate of around 18%.^28^ The study design and details of the questionnaire are reported elsewhere.^28^ Medicare is Australia's universal public health insurance scheme which provides free public hospital care and subsidises a range of services provided out‐of‐hospital and prescription medicines.

### 2.3 Data

Sociodemographic and health variables were derived from the self‐reported baseline questionnaire. Data from the questionnaire were linked to Medicare Benefits Schedule (MBS) claims data (1 January 2003–14 December 2012) provided by Services Australia, and NSW Registry of Births, Deaths and Marriages (RBDM) death registrations data, the latter for censoring purposes. The MBS claims database includes all claims for subsidised medical and diagnostic services provided by registered medical and other practitioners through the MBS, and captures nearly all GP services. For each service claim processed, the MBS data include information on date and the item number for the service. Linkage of baseline data to MBS data was performed at the Sax Institute through deterministic linkage, using an encrypted version of the Medicare number provided directly by Services Australia. Probabilistic linkage was performed by the Centre for Health Record Linkage (CHeReL) for NSW RBDM data. Quality assurance data on the CHeReL data linkage show false positive and negative rates of <0.5% and <0.1%, respectively.^29^

### 2.4 Variables

#### 2.4.1 Individual‐level quality of care outcome variables

Consistent with our conceptual framework, our outcome measures were qualities and types of services received by individuals reflecting high‐quality PC.^1^, ^10^, ^30^ These were: continuity of GP care (yes/no), measured by the usual provider index (UPI),^31^ calculated as the proportion of GP MBS services with the most frequent provider of total GP MBS services and defined as a UPI of 70% or more. As per standard definitions, the UPI was calculated over a 2‐year period and only for participants who used at least four services in that time; any MBS service for a long or prolonged GP consultation (yes/no; which is associated with more problems managed and better outcomes,^32^ including patient enablement^33^ and quality of life^34^); and care planning (yes/no), defined as at least one MBS service for a CD or complex care planning item (including a GP management plan, team care arrangement or review item, shown to support evidence‐based multidisciplinary care and patient education^35^ and regularity of GP care,^36^ with reviews items associated with reduced hospitalisations in some subgroups^37^). See Supporting Information: Table S1 for MBS items codes included in the outcome measures.

#### 2.4.2 Individual‐level explanatory variables

Person characteristics were derived from the 45 and Up baseline questionnaire. Our main exposure variable, SEP, was measured as the highest educational level attained (no school certificate, school certificate, apprenticeship or diploma, university degree, see Supporting Information: File p. 2 for details). To determine need‐adjusted use,^20^ we included the following variables: self‐reported health, physical functioning, and number of self‐reported chronic conditions. We also adjusted for other determinants of health service use^26^ including age, sex, country of birth and marital status (Supporting Information: File p. 2).

#### 2.4.3 Area‐level PC service organisation and delivery characteristics

We constructed measures that, given the available data, best approximate PC service organisation and delivery at the small area level, across the core functions of high‐quality PC systems (Tables 1 and 2). Data sources and construction have been reported in detail elsewhere.^14^ These core functions are: first contact accessibility (including availability, affordability and accommodation), comprehensiveness (provision of care for most needs, e.g., CD and preventative care) and coordination (coordination with other services, skill mix and team‐based care).^1^, ^10^, ^11^ While some of the constructed measures relate to services received by individuals within an area, these measures at an area level can be viewed as a proxy for organisational structures that may be in place that enable the delivery of that core PC function.

**Table 2:** Participants in each education category (%), total and by quartiles of area PC service characteristics.

	No school certificate	School certificate	Apprentice/diploma	University	Total, % (*n*)
*Total participants (%, n)*	*11.7 (31,126)*	*31.8 (84,302)*	*31.8 (84,294)*	*23.0 (60,933)*	*100 (265,083)*
*Area PC service characteristics*					
GP FTE per 1000 URP					
Lowest quartile	35.6	31.2	30.5	23.3	29.7 (78,677)
2nd	25.5	25.7	26.4	25.8	25.9 (68,711)
3rd	20.4	20.5	20.2	19.8	20.2 (53,608)
Highest quartile	18.5	22.6	23.0	31.1	24.2 (64,087)
Average out‐of‐pocket costs per service (AUD)					
Lowest quartile	14.5	11.0	10.3	9.3	10.8 (28,722)
2nd	26.1	26.2	26.3	23.9	25.7 (68,073)
3rd	35.3	35.2	36.2	31.2	34.6 (91,680)
Highest quartile	24.1	27.5	27.2	35.6	28.9 (76,499)
% total GP MBS services bulk‐billed					
Lowest quartile	27.6	30.3	30.6	36.9	31.6 (83,736)
2nd	30.9	31.4	32.2	29.0	31.6 (82,200)
3rd	25.2	25.5	25.6	24.5	25.2 (66,915)
Highest quartile	16.2	12.7	11.6	9.4	12.0 (31,817)
% of total GP MBS services after‐hours					
Lowest quartile	46.8	43.6	42.1	33.0	41.0 (108,758)
2nd	19.9	22.1	23.4	30.6	24.2 (64,204)
3rd	17.1	17.9	17.4	18.7	17.8 (47,190)
Highest quartile	16.1	16.4	17.1	17.7	16.9 (44,822)
Chronic disease care planning and coordination MBS services per 100 self‐reported long‐term conditions					
Lowest quartile	18.2	22.9	24.5	33.3	25.2 (66,798)
2nd	30.7	29.6	28.3	25.5	28.4 (75,228)
3rd	23.4	21.2	20.4	17.1	20.3 (53,685)
Highest quartile	27.6	26.3	26.8	24.1	26.1 (69,224)
Health assessment MBS services per 100 eligible population					
Lowest quartile	18.0	19.3	19.8	22.8	20.0 (53,355)
2nd	17.6	18.6	18.2	21.1	19.0 (50,257)
3rd	22.9	23.5	23.3	25.1	23.7 (62,910)
Highest quartile	41.5	38.5	38.7	30.8	37.1 (98,452)

*Note*: Bulk‐billed refers to where no copayment has been charged to the patient and the provider claims reimbursement for the service directly from Medicare, Australia's universal health insurance scheme. Chronic disease care services involve a comprehensive assessment, management plan and care coordination between healthcare providers and included MBS claims for GP management plans, team care arrangements, and reviews, diabetes and asthma cycles of care item numbers. Self‐reported long‐term conditions modelled from the 2004/2005 National Health Survey. Columns for each variable category for each educational attainment categories sum to 100%. Values in last column gives breakdown by category for each individual variable not stratified by educational attainment. For each variable, total (*n*) sums to 265,083. Missing education category 1.7% (*n* = 4428). First quartile corresponds to 25% of the population in the lowest category, 4th quartile 25% of the population in the highest category; missing's: out‐of‐pocket costs (*n* = 109), bulk‐billing (*n* = 415), after‐hours services (*n* = 109), chronic disease services (*n* = 148), health assessments (*n* = 109) all less than 1%. *χ* ^2^ test of trend *p* < 0.001 for all variable pairs.

Abbreviations: AUD, Australian dollars; FTE, full‐time equivalent; GP, general practitioner; MBS, Medicare Benefits Schedule; URP, usual resident population 2006 census.

All variables were calculated at, and each participant assigned to, the Statistical Area Level 3 (SA3). These areas have similar regional characteristics with populations ranging between 30,000 and 130,000 persons, are often the functional areas of regional cities and large urban transport and service hubs and are the most common geography for which health data are publicly available. As such, these areas represent a reasonable approximation of communities sharing similar characteristics in terms of the organisation and delivery of PC services.

### 2.5 Statistical analysis

Participants were followed for outcomes 2 years after study entry for continuity of care, and 1 year for long consultations and care planning. We included participants if they had at least one Medicare record, were alive at the end of the follow‐up period, resided in NSW, and, for analyses of continuity of care, had at least 3 visits in the follow‐up period.

A series of random‐intercept multilevel logistic regression models (participants nested within SA3 of residence) were fitted for each outcome. Four model specifications were used: (1) to determine if there was significant area‐level variation in outcomes, a random‐intercept model with no explanatory variables; (2) to quantify the association between individual‐level education and outcomes, model 1 additionally included individual‐level education, as well individual‐level demographic and health characteristics to adjust for need and confounders; (3) model 2 further adjusted for each area‐level PC service organisation characteristic separately, to determine the strength of association between a service characteristic and outcome (accounting for individual‐level characteristics); and (4) model 3 additionally adjusted for a cross‐level interaction term between individual‐level education and each area‐level PC service organisation characteristic, to determine whether area service characteristics modified the SEP‐outcome relationship.

Area‐level variation in each outcome was estimated from the variance term (V_A_) by calculating the ICC by the linear threshold model method (ICC = V_A_/(V_A_ + 3.29) and the median odds ratio (MOR = $\text{exp}(0.954\surd V_{A})$.^38^ We report the proportional change in variance (PCV = (V_A_ – V_B_/V_A_) x 100)^38^ as an estimate of the proportion of overall area‐level variation in an outcome explained by the addition of area‐level explanatory variables to the model. Second‐order penalised quasi‐likelihood estimation was used as per Rasbash and colleagues.^39^ Monte Carlo Markov Chain estimation was used to assess model fit and assumptions.

As health service use in Australia varies according to remoteness,^14^ analyses were stratified by categories of geographical remoteness (major cities, inner regional, outer regional/remote) based the 2006 Access and Remoteness Index of Australia (ARIA+),^40^ which classifies all of Australia based upon road distance to the nearest city or town in each five classes based on population size. Analyses examining after‐hours care as an area‐level explanatory variable were restricted to major cities and inner regional areas, as a substantial proportion of after‐hours care in remote Australia is provided through outpatient and emergency department. Sensitivity analyses were performed, repeating the main analysis but including those who died in the follow‐up period.

Analyses were undertaken using Stata (StataCorp; Version 14.1) in the Secure Unified Research Environment, a secure remote‐access computer facility for analysis of linked data. Multilevel analysis was performed using the runmlwin add‐on.^41^

## 3 RESULTS

### 3.1 Sample characteristics

After excluding those who had an invalid death date or died in the follow‐up period (*n* = 320), did not have an MBS service (*n* = 1548), for whom data were unavailable at the time of analysis (*n* = 41) or were unable to be assigned to an SA3 (*n* = 161), 265,083 participants were included in the study. Of these, 11.7% had no school certificate, 31.8% completed a school certificate, 31.8% had completed an apprenticeship or diploma and 23% had completed a tertiary‐level qualification (Table 2). The mean age was 62.7 years (SD 11.2), 46% were male, over 80% rated their health as good, very good or excellent and 73% had at least one chronic condition (Supporting Information: Table S2). Individuals with low education were more likely to live in areas with fewer GPs per capita and after‐hours services, lower out‐of‐pocket costs (OPC), and more bulk‐billing and CD services than those with higher education (Table 2). The proportion of participants with quality‐of‐care outcomes was higher among people with lower compared with higher education, and in areas with lower OPC, more bulk‐billing and after‐hours and CD services (Table 3).

**Table 3:** Quality of care outcomes by education and area PC service characteristics (%, *n*).

	Continuity of care	Long consultations	Care planning
*Education*			
No school certificate	59.9 (17,298/28,881)	43.5 (13,550/31,126)	29.6 (6985/23,636)
School certificate	56.2 (42,556/75,689)	41.3 (34,800/84,299)	22.5 (13,396/59,553)
Apprentice/diploma	54.3 (40,193/74,046)	39.9 (33,631/84,294)	19.6 (11,356/57,979)
University	50.3 (25,577/50,842)	40.2 (24,506/60,933)	13.1 (5188/39,520)
*Area PC service characteristics*			
GPs per capita			
1st quartile	54.0 (37,022/68,546)	37.7 (29,625/78,677)	20.6 (11,338/55,148)
2nd quartile	55.9 (33,958/60,745)	41.4 (28,477/68,711)	20.5 (9854/48087)
3rd quartile	56.3 (26,965/47,905)	40.6 (21,770/53,607)	21.6 (7966/36959)
4th quartile	53.5 (30,110/56,292)	44.6 (28,556/64,085)	19.8 (8657/43667)
Out‐of‐pocket costs (AUD)			
1st quartile	59.8 (15,914/26,609)	43.1 (12,371/28,721)	26.3 (5294/20,116)
2nd quartile	54.6 (33,160/60,725)	43.9 (29,915/68,073)	22.2 (10,532/47,338)
3rd quartile	53.3 (43,227/81,082)	40.2 (36,832/91,680)	22.1 (14,207/64,191)
4th quartile	54.9 (35,698/64,976)	38.3 (29,261/76,497)	14.9 (7772/52,140)
Bulk‐billing (% services)			
1st quartile	55.9 (39,717/71,017)	37.0 (30,995/83,735)	14.8 (8489/57,468)
2nd quartile	51.5 (37,529/72,905)	41.3 (33,932/82,199)	22.6 (12,992/57,409)
3rd quartile	54.9 (32,756/59,655)	44.2 (29,582/66,915)	22.5 (10,427/46,396)
4th quartile	60.4 (17,856/29,548)	43.2 (13,757/31,816)	26.3 (5863/22,321)
After‐hours care (% services)			
1st quartile	53.0 (23,177/43,761)	36.1 (18,082/50,105)	22.3 (7837/35,119)
2nd quartile	55.6 (27,863/50,098)	43.4 (24,809/57,142)	17.1 (6685/39,188)
3rd quartile	56.8 (22,025/38,778)	46.4 (19,881/42,849)	22.6 (6698/29,620)
4th quartile	57.4 (23,277/40,563)	43 (19,266/44,821)	22.3 (6888/30,915)
Chronic disease care planning and coordination (per 100 chronic conditions)			
1st quartile	55.2 (31,624/57,338)	–	–
2nd quartile	54.7 (35,767/65,330)	–	–
3rd quartile	56.0 (27,196/48,561)	–	–
4th quartile	53.7 (33,393/62,130)	–	–
Health assessments (per 100 eligible population)			
1st quartile	55.1 (25,887/46,968)	–	–
2nd quartile	56.6 (25,052/44,280)	–	–
3rd quartile	57.5 (31,436/54,694)	–	–
4th quartile	52.2 (45,624/87,450)	–	–
Total % (*n*)	54.8 (128,055/233,488)	40.9 (108,428/265,080)	20.6 (37,815/183,861)

*Note*: First quartile corresponds to 25% of the population in the lowest category, 4th quartile 25% of the population in the highest category. Bulk‐billed refers to where no copayment has been charged to the patient and the provider claims reimbursement for the service directly from Medicare, Australia's universal health insurance scheme. For analyses of continuity of care, those who died in the 2nd year or had less than four GP MBS services during follow‐up were excluded (*n* = 31,595). Additional exclusions included, for analyses of care planning participants without a chronic disease (*n* = 81,222), and for analyses of long consultations outlier observations (*n* = 3).

Abbreviations: GP, general practitioner.

### 3.2 Association of individual‐level education and area PC service characteristics with quality of care

For a given level of need and accounting for area variation, low‐education individuals were more likely to have continuity of care (e.g., university vs. no school certificate in major cities, odds ratio [OR] 0.88, 95% confidence interval [CI] [0.83, 0.93]) and care planning (e.g., major cities 0.66 [0.61, 0.71]), but less likely to have a long consultation (e.g., inner regional 1.11 [1.05, 1.16], Supporting Information: Table S3). Patterns of association were found whether in major cities or more remote locations.

Fitting of the empty multilevel model (i.e., with no explanatory variables) and model fit statistics confirmed area‐level variation for all outcomes (MOR: continuity of care, 1.16–1.40, long consults, 1.18–1.37, care planning, 1.48–1.44, *p* < 0.001, Supporting Information: Table S4). For all outcomes in all regions, area‐level PC service characteristics explained part of the between‐area variation as evidenced by a reduction in PCV after the addition of area‐level variables to models adjusted for individual characteristics (Supporting Information: Tables S5–S7).

**Figure 2:**
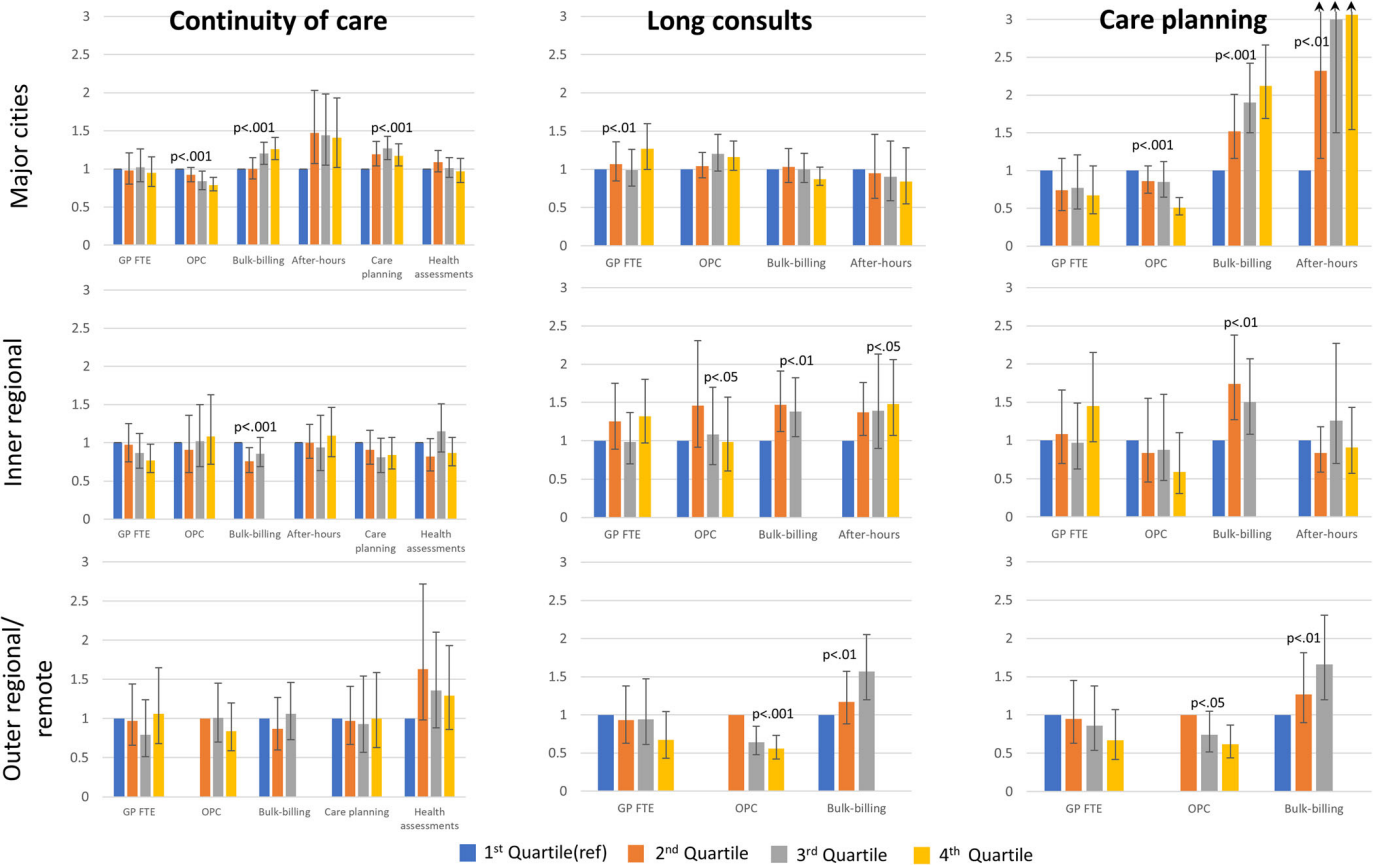
Association of area‐level PC service characteristics and quality of care outcomes, separately by region (odds ratio and 95% confidence intervals). Wald joint test of significance shown. Models adjusted for individual sociodemographic and need variables. Bulk‐billing refers to where no copayment has been charged to the patient and the provider claims reimbursement for the service directly from Medicare, Australia's universal health insurance scheme. FTE, full‐time equivalent (per capita); GP, general practitioner; OPC, out‐of‐pocket costs; OR, odds ratio; PC, primary care.

In major cities alone, people who lived in areas with more bulk‐billing, CD services and fewer OPC were more likely to have continuity of care, accounting for individual characteristics (highest quartile compared with lowest: OPC, 0.79 [0.71, 0.89], PCV 34%; bulk‐billing, 1.26 [1.12, 1.41], 41%; CD care 1.17 [1.04, 1.33], 31%; Figure 2, Supporting Information: Table S5). However, in regional areas, people living in areas with more bulk billing were less likely to have continuity of care (0.86 [0.69, 1.07], 19%). More bulk‐billing and after‐hours services and fewer OPC were similarly associated with care planning (all regions) and long consultations (regional areas only; Figure 2, Supporting Information: Tables S6–S7). No clear pattern of association was evident for GP availability and outcomes.

### 3.3 Effect modification by area‐level PC service characteristics

ORs for cross‐level interaction terms are interpreted as the effect of an increasing level of the area‐level PC characteristic on the odds of the outcome for that education category compared with the lowest education category (Table 4).

**Table 4:** Continuity of care and long consultations, cross‐level effect modification: Odds ratio and 95% confidence interval for area PC service characteristics (continuous) and as interaction with education, separately by region.

	Continuity of care			Long consultations		
	Cities	Inner regional	Outer regional/remote	Cities	Inner regional	Outer regional/remote
PC characteristic and interaction terms	OR (95%CI)	OR (95%CI)	OR (95%CI)	OR (95%CI)	OR (95%CI)	OR (95%CI)
*GP FTE*						
No school certificate × GP FTE	–	1	1	1	1	1
School certificate × GP FTE	–	1.009 (0.775–1.313)	0.749 (0.544–1.030)	0.962 (0.709–1.305)	0.834 (0.645–1.079)	1.094 (0.802–1.492)
Apprentice/diploma × GP FTE	–	0.858 (0.660–1.117)	0.864 (0.620–1.204)	0.890 (0.656–1.207)	0.844 (0.653–1.090)	1.144 (0.828–1.579)
University × GP FTE	–	1.068 (0.799–1.427)	0.626 (0.425–0.923)	0.891 (0.662–1.199)	0.836 (0.633–1.105)	1.326 (0.912–1.929)
*p* Value	–	0.214	0.080	0.749	0.536	0.504
*OOP expenses*						
No school certificate × OPC	1	1	1	1	–	1
School certificate × OPC	0.981 (0.965–0.997)	0.982 (0.961–1.003)	1.021 (0.992–1.050)	0.988 (0.973–1.002)	–	0.992 (0.966–1.020)
Apprentice/diploma × OPC	0.980 (0.964–0.996)	0.996 (0.975–1.017)	1.035 (1.005–1.065)	0.991 (0.976–1.006)	–	0.998 (0.971–1.026)
University × OPC	0.988 (0.972–1.004)	0.984 (0.962–1.006)	1.027 (0.993–1.062)	0.987 (0.973–1.002)	–	1.025 (0.993–1.058)
*p* Value	**0.028**	0.156	0.134	0.336	–	0.112
*Bulk‐billing*						
No school certificate × bulk‐billing	1	1	1	–	1	1
School certificate × bulk‐billing	1.006 (1.001–1.012)	1.005 (0.999–1.012)	0.998 (0.991–1.005)	–	1.006 (1.000–1.012)	1.002 (0.995–1.008)
Apprentice/diploma × bulk‐billing	1.008 (1.003–1.013)	1.002 (0.996–1.008)	0.995 (0.988–1.001)	–	1.003 (0.997–1.009)	1.002 (0.995–1.008)
University × bulk‐billing	1.006 (1.000–1.011)	1.004 (0.998–1.011)	0.995 (0.987–1.003)	–	1.003 (0.997–1.010)	0.995 (0.987–1.003)
*p* Value	**0.030**	0.241	0.357	–	0.279	0.151
*After‐hours care*						
No school certificate × after‐hours	1	–	–	1	1	–
School certificate × after‐hours	1.012 (0.992–1.033)	–	–	1.005 (0.987–1.024)	0.988 (0.970–1.006)	–
Apprentice/diploma × after‐hours	1.012 (0.992–1.033)	–	–	1.011 (0.992–1.030)	0.985 (0.967–1.003)	–
University × after‐hours	0.996 (0.975–1.016)	–	–	1.005 (0.986–1.024)	0.970 (0.951–0.989)	–
*p* Value	**0.054**	–	–	0.661	**0.012**	–
*CD care*						
No school certificate × CD care	1	1	1	–	–	–
School certificate × CD care	1.006 (0.999–1.013)	0.999 (0.994–1.005)	0.998 (0.993–1.002)	–	–	–
Apprentice/diploma × CD care	1.010 (1.003–1.017)	1.000 (0.995–1.006)	0.996 (0.992–1.001)	–	–	–
University × CD care	1.007 (1.000–1.014)	1.001 (0.995–1.007)	0.997 (0.992–1.003)	–	–	–
*p* Value	**0.041**	0.89	0.542	–	–	–

*Note*: Models additionally adjusted for individual age, sex, marital status, country of birth and need variables (self‐reported health, number of chronic conditions, physical functioning). Continuous variable and interaction term with education for each area PC characteristic added separately. *p* Value, Wald test of joint significance for the addition of cross‐level interaction term. Significant terms bolded. Terms not reported did not have a monotonic relationship as a categorical variable with outcome. After‐hours care not tested in outer regional/remote areas due to unreliability of this estimate in this region. CD Care not tested with long consultations given uncertain interpretation.

Abbreviations: CD, chronic disease; CI, confidence interval; FTE, full‐time equivalent; GP, general practitioner; OPC, out‐of‐pocket cost; OR, odds ratio; PC, primary care.

**Figure 3:**
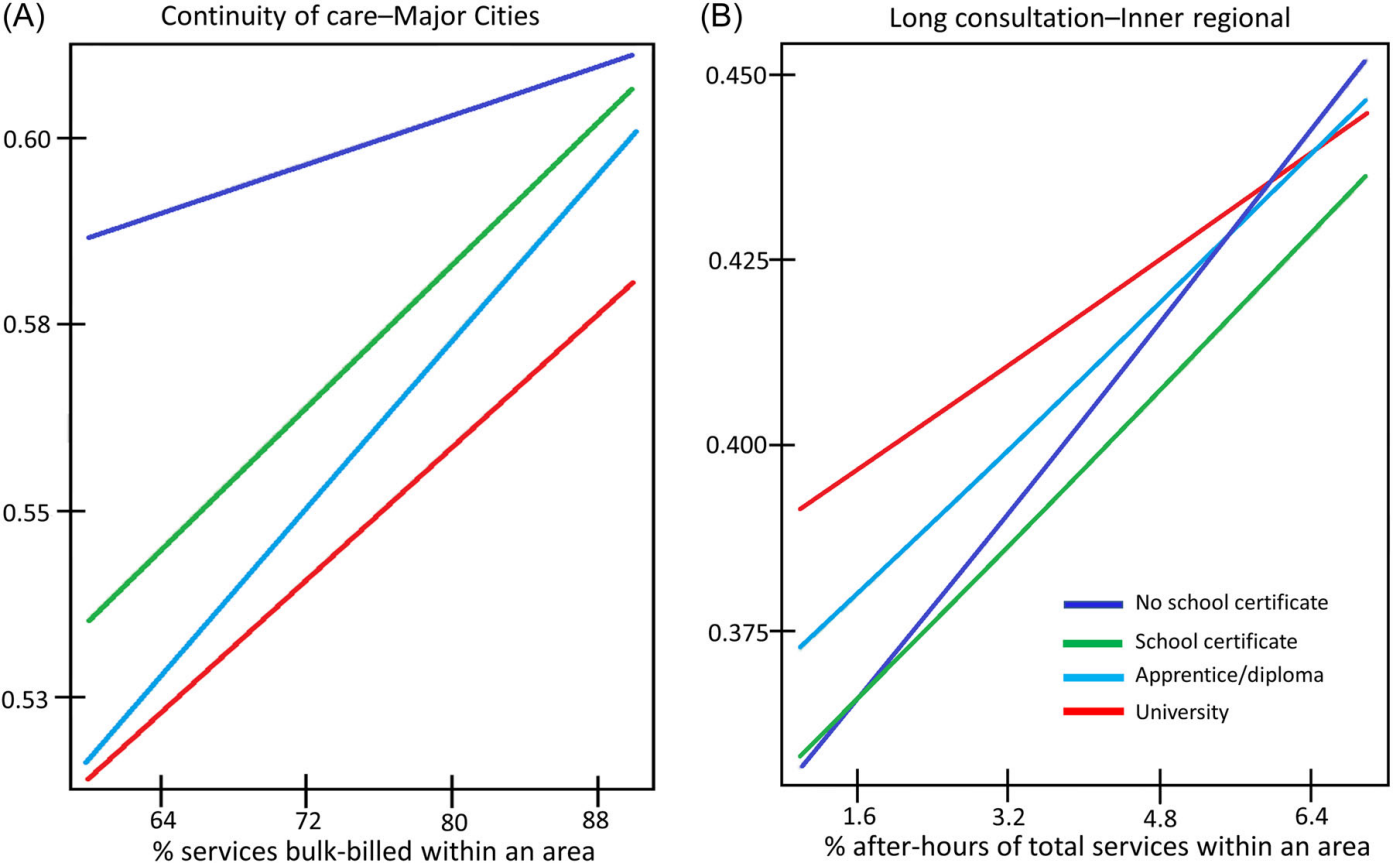
Cross‐level effect modification, (A) predicted probabilities of continuity of care by education and bulk‐billing, major cities and (B) predicted probabilities of a long consultation by education and after‐hours care, inner regional. Models additionally adjusted for individual age, sex, marital status, country of birth and need variables (self‐reported health, number of chronic conditions, physical functioning). Values for prediction for after‐hours care ranged from 3% to 12% of all services and for bulk‐billing ranged from 60% to 90% of all services. Interaction terms all significant to the *p* < 0.05 level. Bulk‐billing refers to where no copayment has been charged to the patient and the provider claims reimbursement for the service directly from Medicare, Australia's universal health insurance scheme.

In major cities, living in areas with more bulk‐billing, CD services and fewer OPC was associated with larger increases in the likelihood of receiving continuity of care among high‐education individuals than low‐education (university vs. no school certificate, interaction term for: OPC 0.988 [0.912–1.004], bulk‐billing, 1.006 [1.000–1.011]), CD services 1.007 [1.000–1.014], Table 4, Figure 3). By contrast, in inner regional locations more after‐hours services within areas was associated with larger increases in the likelihood of long consultations among low‐education individuals than high‐education (0.970 [0.951–0.989]). As shown in Figure 3, the pro‐high education association with long consultations reverses in areas with the highest quartile of after‐hours services; low‐education individuals were more likely to have a long consultation compared with high‐education. No other associations with found in models with interaction terms, including models where the outcome was care planning (Supporting Information: Table S8).

## 4 DISCUSSION

This study shows that organisation and delivery of PC services at the small area level modifies the relationship between individual SEP and quality‐of‐GP care. In major cities, people of all education levels who lived in areas with more bulk‐billing, after‐hours or chronic disease services and fewer OPC, were more likely to have continuity of care. However, these service characteristics were associated with larger increases in the odds of continuity of care among high‐education individuals, such that they approximate that of their low‐education counterparts. By contrast, in regional areas, the increase in the likelihood of a long consultation in areas with more after‐hours services was larger for low‐ compared with high‐education individuals. GP availability in isolation was not associated with a relative benefit for low‐education individuals, regardless of geographical location.

The finding that people of low‐SEP were more likely to have continuity of care (having accounted for need) is consistent with an Australian study which found people of low income were more likely to report being affiliated with single GP (self‐reported)^42^ as well as findings from other countries with universal health insurance.^43^, ^44^ To our knowledge, this is the first study to examine the association of area‐level service organisation with individual socioeconomic variation in quality‐of‐GP care. Given the observational nature of the study, the direction and mechanisms underlying the associations found here are uncertain and requires further exploration. Nevertheless, a possible interpretation of the findings is that measures intended to contain costs of care for individuals (e.g., bulk‐billing), accommodate patient preferences and needs (e.g., after‐hours services) or increase chronic disease care planning and coordination may support quality‐of‐care, as measured here. However, the findings also indicate that in major cities increasing these measures (i.e., bulk‐billing or after‐hours access) locally may produce only marginal gains for low‐SEP groups in terms of continuity of care. Conversely in regional areas, a possible interpretation is that increasing after‐hours services at the small area level may support access to long consultations, especially disadvantaged individuals who typically have greater healthcare needs which are currently under‐serviced. Again, further exploration of the mechanisms underlying this association is required to better understand appropriate policy responses based on this finding.

Using a large sample linked to MBS service data and a multilevel framework for analysis allowed for investigation of nested levels of associations and, more importantly, how these levels relate to each other in shaping use of health services. However, study limitations should be kept in mind when interpreting the findings. First, the measures of PC service organisation used are the best approximation of these characteristics given data available at this geographical scale. Ideally, measures would reflect the extent to which most services in an area incorporate aspects of coordination (e.g., team‐based care, role substitution, skill‐mix), comprehensiveness (e.g., programmes for chronic diseases or maternal/child health or scope of practice scores for providers^45^) or accommodation (e.g., appointment systems, walk‐in facilities). Such comprehensive data are currently unavailable. Further, the geographical unit of analysis was also a best approximation, and as such associations may be underestimated. Second, variation in outcomes may also be explained by variation at the practice‐ or provider level. However, we were unable to account for this with the data available. Third, the 45 and Up study is not representative of the NSW population,^46^ and while representativeness is not necessary for internal validity (i.e., relative effect estimates),^46^ patterns of association may differ for other age groups or in other settings. Fourth, due to limited sample size in outer regional/remote areas, there is uncertainty in our estimates of association between area‐level exposures and outcomes. Finally, we included measures of healthcare need as best able given the available data. However, they are unlikely to have captured true levels of need within the study population with subsequent under‐adjustment in models, potentially accounting for some of the pro‐low education association with outcomes.

While a possible interpretation of the findings is that current initiatives are working well for the socioeconomically disadvantaged in major cities, this is not to suggest that their healthcare needs are fully met. Australia's funding arrangement for GP services is mostly fee‐for‐service,^47^ with limited incentives for promoting equitable use and quality of care.^48^ Alternative funding arrangements or models of care may be required to improve access to quality care for low‐SEP individuals. For example, the patient‐centred medical home has been recently trialled in Australia,^49^ while the Aboriginal community‐controlled health sector have provided comprehensive PC services for decades. Both differ substantially in their models of care and funding arrangements to most PC services in Australia and have been shown to improve outcomes, particularly for those medically underserved or of low‐SEP.^50^, ^51^

While we were unable to establish the direction of associations between area‐level PC characteristics and outcomes, our findings indicate that building on existing measures to support after‐hours services in regional locations may encourage longer GP consultations, especially to disadvantaged individuals with multiple and complex care needs. Should such initiatives reach a similar threshold as found in major cities, alternative models of care or funding arrangements may be required for additional equity gains. Moreover, unintended negative consequences should be monitored, such as excessive workforce turnover and reliance on fly‐in/locum GPs which can compromise continuity of care,^52^ especially for disadvantaged individuals.

A significant focus of PC policy in Australia has been incentives and restrictions designed to address workforce shortages in outer metropolitan or more remote areas.^53^ More recently, these policies have been refined to focus on all communities with the greatest recruitment and retention needs.^53^, ^54^ Yet our study based on data from a cohort in NSW did not find an association between local GP availability and equitable quality of care. However, these initiatives are likely to reduce urban‐rural differences in quality of care and may favour low‐SEP individuals. However, without greater restrictions on where GPs can practice (and how much they can charge), as well as incentives to support equitable high‐quality care, further benefits for low‐SEP individuals may not be realised. In this regard, qualitative work may provide greater understanding on the relationship between local availability of GPs and quality of care and offer practical, and acceptable, policy solutions.

Equitable access to and quality of PC is considered and theorised to be a key mechanism by which health equity can be achieved; yet this is rarely directly investigated. While some of the area‐level and outcome measures may be specific to the Australian context, this study offers other jurisdictions internationally a conceptual and analytical approach for measuring and evaluating equity in PC systems. Further to that end, accounting for the interplay of system‐level factors operating at the area‐, practice‐ and provider‐level (which was unable to be addressed here) is an important and necessary next step.

## 5 CONCLUSION

This study has identified potential opportunities for improving care for those who need it most by strengthening specific aspects of geographical service organisation at the small area level. In major cities, service characteristics were not associated with better healthcare equity, and further gains likely require alternative approaches to how care is provided and funded. In regional locations increasing levels of after‐hours services at the small area level was associated with a specific benefit for low‐SEP individuals and may be a potential avenue for improvement. However, further exploration of the mechanism underlying this observation is required. Improved data measuring the organisation and delivery of PC are required so that the impact of policy initiatives and programme interventions on healthcare equity can be comprehensively assessed.

## AUTHOR CONTRIBUTIONS

Danielle C. Butler, Sarah Larkins, and Rosemary J. Korda conceived and designed the analysis. Danielle C. Butler completed data analysis and drafted the manuscript. All authors revised the work for intellectual content and approved the final version of the manuscript.

## CONFLICT OF INTEREST STATEMENT

The authors declare no conflict of interest.

## ETHICS STATEMENT

Ethics approval for this project was obtained from the NSW Population and Health Services Research Ethics Committee (HREC/13/CIPHS/8), the University of Western Sydney Ethics Committee (H9835) and the Australian National University Human Research Ethics Committee (2011/703). Ethics approval for the 45 and Up Study was granted by the University of New South Wales Human Research Ethics Committee. The 45 and Up Study participants consented to data linkage at baseline. Linkage of the MBS data is performed under approvals from the approval committee of Services Australia and the Australian Government Department of Health.

## Supporting information

Supporting information.

Supporting information.

## Data Availability

The data that support the findings of this study are available from the Sax Institute, NSW but restrictions apply to the availability of these data, which were used under license for the current study, and so are not publicly available. Data from the Sax Institute's 45 and Up Study are available for approved projects to approved researchers (www.saxinstitute.org.au).
