# Supplementary material for: Association of individual‐socioeconomic variation in quality‐of‐primary care with area‐level service organisation: A multilevel analysis using linked data
Source: J Eval Clin Pract. 2023 Mar 9;29(6):984–97. doi: 10.1111/jep.13834 (PMC10946916; doi:10.1111/jep.13834)
Supplement: Supplementary file 2 — Supporting information. [file JEP-29-984-s001.pdf]

Supplementary materials

Supplementary table S1: Item numbers for GP services

MBS item groups and name of group	Specific item numbers within item group	Notes on item numbers
A1-GP attendances	3, 23, 36, 44<br><br>Remainder A1 attendances=4, 20, 24, 35, 37, 43, 47, 51	Items for services in consulting rooms<br>Items relate to consultations at residential care OR other than residential care/consulting rooms
A2-MPs other than GP	52,53,54,57<br><br>Remainder of A2 attendances=58–60, 65, 92, 93, 95, 96	Items for services in consulting rooms<br>Items relate to consultations at residential care OR other than residential care/consulting rooms
A11-GP	597, 599	Urgent after-hours care
A11-MP other than GP	598, 600	Urgent after-hours care
A14-heath Assessments	701, 703, 705, 707	
A14-health assessments for Aboriginal and Torres Strait Islander peoples	715	
A15-GP management plans	721, 723, 729, 731, 732	Care plans, care plan reviews and team care arrangements
A15-case conference	735, 739, 743, 747, 750, 758	
A17	900 and 903	Medication reviews
A18-GP, cervical screening	2497, 2501, 2503, 2504, 2506, 2507, 2509	Incentive for overdue pap smears (>4 years since last pap)
A18-GP asthma	2546, 2547, 2552, 2553, 2558, 2559	cycle of care
A18-GP diabetes	2517, 2518, 2521, 2522, 2525, 2526	cycle of care
A19-non-referred cervical screening	2598, 2600, 2603, 2606, 2610, 2613, 2616	As per A19 for non-VR MPs
A19-non-referred, asthma	2664, 2666, 2668, 2673, 2675, 2677	As for A18 for non-VR MPs
A19-non-referred, diabetes	2620, 2622, 2624, 2631, 2633, 2635	As for A18 for non-VR MPs
A20-GP mental health care plans	2700, 2701, 2712, 2713, 2715, 2717, 2721, 2723, 2725, 2727	Preparation and review of mental health care plans
A22-after hours (GP attendances)	5000, 5003, 5010, 5020, 5023, 5028, 5040, 5043, 5049, 5060, 5063, 5067	Includes consultation room, residential facility and other. At consultation rooms bolded
A23-after hours (non-referred)	5200, 5203, 5207, 5208, 5220, 5223, 5227, 5228, 5260, 5263, 5265, 5267	As per A22 for non-VR MPs

Notes:

1. GP, general practitioner; MP, medical practitioner; VR vocationally registered.
2. All item numbers were included for the continuity of care outcome measure. The specific item numbers included for length of consultation were 36, 44, 54, and 57. The specific item numbers for care planning included all those listed under A15 GP management plans.
3. Medical practitioners (MPs) in this case refer to medical doctors who provide non-referred services but are not vocationally registered as a GP (i.e. have not undertaken specialist training in General Practice or other equivalent recognised training program).

Derivation of main exposure variable education.

With respect to education in the 45 and Up Study questionnaire, participants were asked “What is the highest qualification you have completed?” with the following response options: no school certificate or qualifications, school or intermediate certificate, higher school or leaving certificate, trade/apprenticeship, certificate/diploma and university degree or higher.

We recoded these to four categories for analysis as follows: no school certificate (no school certificate or qualifications), school certificate (school or intermediate certificate), apprenticeship/diploma (trade/apprenticeship, certificate/diploma) and university (university degree or higher).

Data Sources for area PHC service characteristics

PHC service organisation and delivery variables were derived from the following data sources[1]: the Australian Institute of Health and Welfare (AIHW) health workforce survey (2007); Public health information development unit (PHIDU) Social Health Atlas of Australia (2010, 2011, 2014); Medicare Benefits Schedule (MBS) service claims data 2008–2012, Services Australia; Australian Bureau of Statistics (ABS) 2006 Census; Access and Remoteness Index of Australia plus (ARIA+), 2006, National Centre for Social Applications of Geographical Information Systems (GISCA), Adelaide; National Health Performance Authority (NHPA), 2011-2012.

1. Butler DC, Jorm LR, Larkins S, Humphreys J, Desborough J, Korda KJ. Examining area-level variation in service organisation and delivery across the breadth of primary healthcare. Usefulness of measures constructed from routine data. *PloS one*. 2021;16(12):e0260615. doi:10.1371/journal.pone.0260615

Supplementary table S2. Sample characteristic, individual-level variables by educational attainment (%) and for total sample

Variable	Educational attainment					
	No school certificate	School certificate	Apprentice/ diploma	University	Missing	Total for row category %(n)
Educational attainment						
Total %(n)	11.7(31,126)	31.8(84,302)	31.8(84,294)	23.0(60,933)	1.7(4,428)	100(265,083)
Sex						
Male	42.1	36.1	55.2	50	48.6	46.4(122,893)
Female	57.3	63.9	44.8	50	51.5	53.6(142,190)
Age						
45–54	16.5	24.4	31.6	40.1	14.4	29.2(77,397)
55–64	27.4	32.9	31.9	34.8	22.6	32.2(85,342)
65–74	28.9	23.7	21.5	15.7	25.4	21.8(57,734)
75–84	21.8	15.3	12.7	8	28.8	13.8(36,516)
85 plus	5.4	3.8	2.3	1.5	8.8	3.1(8,082)
Country of birth						
Australia/NZ	75.9	80.8	76.9	72.5	64.8	76.8(203,629)
Europe/ N.America	18.6	13.4	17.7	16.8	20.9	16.3(43,154)
Asia	2.3	2.6	2.3	6.5	4	3.4(9,031)
Africa/Mid. East	1.1	1.5	1.3	2.7	1.7	1.7(4,397)
Other	0.5	0.8	0.9	0.8	0.9	0.08(2,145)
Marital status						
Not married/ not de facto	32.6	26.2	22.3	21	33.5	24.6(65,288)
Married/de facto	66.8	73.3	77.1	78.5	64.3	74.7(198,185)
Self-rated health						
Excellent	7.4	12.2	14.1	22.4	10.1	14.6(38,575)
Very good	25.6	35	36.8	40.8	24.6	36.5(94,481)
Good	36.3	34.5	33.8	26.5	32.1	32.6(86,451)
Fair	20.5	12.3	10.7	6.9	17.5	11.6(30,644)
Poor	4.8	2.2	1.8	1	4	2.1(5,575)
Chronic conditions						
none	20.7	25.4	27.3	31.2	25.2	26.8(70,991)
1–2	49.9	52	52.6	53	50.1	52.1(198,116)
3 or more	29.4	22.6	20.2	15.8	24.7	21.1(55,976)
Physical functioning						
No limitation	18.9	26.5	30	39.4	19.2	29.5(78,323)
Minor limitation	15.4	23.2	26.9	30.2	14.5	24.9(66,072)
Moderate limitation	21.4	22.5	21.6	17.3	16.7	20.8(55,097)
Severe limitation	21.8	12.9	10.3	5.5	16.5	11.5(30,367)

Notes: Abbrev. N, number; %, percentage. Columns for each variable category for each educational attainment categories sum to 100%. Values in last column gives break down by category for each individual variables for the total sample, not stratified by educational attainment. For each variable, total (n) sums to 265,083 and percent sums to 100% including missings. Chi-squared test for trend with education p<.001 all variables. Missing: age<1%, country of birth 1%, marital status 0.6%, self-rated health 3.5%, physical functioning 13.3%, continuity of care 0.1%, care planning 30.6%.

Supplementary table S3. Fixed effects for the association of education with quality-of-care outcomes, by region

	Cities	Inner regional	Outer regional/ remote
	OR (95%CI)	OR (95%CI)	OR (95%CI)
**Continuity of care**			
No school certificate(ref)	1	1	1
School certificate	0.98 (0.93–1.03)	0.98 (0.93–1.03)	1.02 (0.97–1.08)
Apprentice/ diploma	0.94 (0.90–1.00)	0.94 (0.90–1.00)	0.92 (0.87–0.97)
University	0.88 (0.83–0.93)	0.88 (0.83–0.93)	0.87 (0.82–0.92)
**Long consults**			
No school certificate(ref)	1	1	1
School certificate	1.00 (0.95–1.05)	0.99 (0.95–1.04)	0.97 (0.93–1.02)
Apprentice/ diploma	1.05 (1.00–1.10)	1.05 (1.00–1.10)	1.02 (0.97–1.08)
University	1.05 (0.99–1.10)	1.11 (1.05–1.16)	1.09 (1.02–1.15)
**Care planning**			
No school certificate(ref)	1	1	1
School certificate	0.86 (0.81–0.92)	0.85 (0.80–0.91)	0.89 (0.83–0.95)
Apprentice/ diploma	0.80 (0.75–0.85)	0.78 (0.73–0.83)	0.78 (0.72–0.83)
University	0.66 (0.61–0.71)	0.61 (0.57–0.66)	0.62 (0.57–0.68)

Notes: OR, odds ratio; CI, confidence interval. Adjusted for sociodemographic (educational, age, sex, country of birth, marital status) and need (self-rated health status, number of chronic disease, physical functioning) variables. Wald joint test of significance for education p<.001 for all outcomes and regions, except long consults in major cities (p<.05).

Supplementary table S4. Between-area variation in odds of quality of GP care outcomes

	Long consults		Continuity of care		Care planning	
	Null model	Model 2	Null model	Model 2	Null model	Model 2
**Cities**						
ICC	0.009	0.013	0.007	0.007	0.049	0.039
PCV (%)	na	-44	na	1.5	na	22
MOR	1.18	1.21	1.16	1.16	1.48	1.41
**Inner regional**						
ICC	0.027	0.031	0.017	0.019	0.049	0.048
PCV (%)	na	-16	na	-8	na	4
MOR	1.33	1.36	1.26	1.27	1.48	1.47
**Outer regional**						
ICC	0.033	0.041	0.037	0.037	0.043	0.044
PCV (%)	na	-24	na	-0.4	na	-2.1
MOR	1.37	1.43	1.40	1.40	1.44	1.45

Notes:

1. ICC: intra-class coefficient; PCV: proportional change in variance (reported change from null model), MOR median odds ratio; na, not applicable.
2. Null model, random intercept only, no covariates; Model 2 adjusted for education, age, sex, country of birth, marital status, self-rated health, chronic disease and physical functional limitation.
3. Wald test that between-area variance for the null model is non zero (i.e. δ_u^2 =0) p<.001 for all outcomes.

Supplementary Table S5. Association of PC characteristics with continuity-of-care, odds ratio and 95% confidence interval, by region

	GP FTE	*Out-of-pocket costs*	*Bulk-billing*	*After-hours care*	*CD care*	*Health assessments*
	**OR (95%CI)**	**OR (95%CI)**	**OR (95%CI)**	**OR (95%CI)**	**OR (95%CI)**	**OR (95%CI)**
**Cities**						
1st quartile (ref)	1	1	1	1	1	1
2nd quartile	0.98 (0.80–1.21)	0.92 (0.83–1.02)	1.00 (0.87–1.15)	1.47 (1.07–2.03)	1.19 (1.04–1.36)	1.09 (0.96–1.24)
3rd quartile	1.02 (0.83–1.26)	0.84 (0.73–0.97)	1.20 (1.06–1.35)	1.44 (1.05–1.98)	1.27 (1.12–1.43)	1.01 (0.89–1.15)
4th quartile	0.95 (0.77–1.16)	0.79 (0.71–0.89)	1.26 (1.12–1.41)	1.41 (1.02–1.93)	1.17 (1.04–1.33)	0.97 (0.82–1.14)
p-value for term	0.694	**<.001**	**<.001**	0.127	**<.001**	0.427
ICC	0.007	0.005	0.004	0.006	0.005	0.007
PCV (%)	4	34	41	13	31	8
MOR	1.16	1.13	1.12	1.15	1.13	1.15
**Inner regional**						
1st quartile (ref.)	1	1	1	1	1	1
2nd quartile	0.97 (0.75–1.25)	0.91 (0.61–1.36)	0.76 (0.61–0.94)	1.00 (0.80–1.24)	0.91 (0.72–1.16)	0.82 (0.63–1.05)
3rd quartile	0.87 (0.67–1.12)	1.02 (0.69–1.50)	0.86 (0.69–1.07)	0.94 (0.64–1.36)	0.81 (0.61–1.06)	1.15 (0.88–1.51)
4th quartile	0.77 (0.61–0.98)	1.08 (0.72–1.63)	–	1.09 (0.82–1.46)	0.84 (0.66–1.07)	0.87 (0.70–1.07)
p-value for term	0.169	0.616	**0.036**	0.899	0.357	0.069
ICC	0.016	0.017	0.015	0.018	0.017	0.015
PCV (%)	15	6	19	3	10	21
MOR	1.24	1.26	1.24	1.27	1.25	1.24
**Outer regional**						
1st quartile (ref.)	1	–	1	–	1	1
2nd quartile	0.97 (0.66–1.44)	ref	0.87 (0.60–1.27)	–	0.97 (0.67–1.41)	1.63 (0.98–2.72)
3rd quartile	0.79 (0.51–1.24)	1.01 (0.70–1.45)	1.03 (0.73–1.46)	–	0.93 (0.57–1.54)	1.36 (0.88–2.10)
4th quartile	1.06 (0.68–1.65)	0.84 (0.59–1.20)	–	–	1.00 (0.63–1.59)	1.29 (0.86–1.93)
p-value for term	0.739	0.532	0.659	**–**	0.993	0.293
ICC	0.035	0.035	0.036	–	0.037	0.031
PCV (%)	4	5	3	–	0	16
MOR	1.4	1.39	1.4	–	1.41	1.37

Notes: PC, primary care; OR, odds ratio; CI, confidence interval; GP, general practitioner; FTE, full-time equivalent; OPC, out-of-pocket costs; ICC, Intra-class coefficients; PCV, proportional change in variance; MOR, median odds ratio. Odds ratio and 95% confidence intervals shown, Wald joint test of significance shown. Significant terms bolded. 1st quartile corresponds to 25% of the population in the lowest category, 4th quartile 25% of the population in the highest category. Models adjusted for individual sociodemographic and need variables. PCV reported change from model adjusted for sociodemographic and need variables (without area-level variable). Bulk-billing refers to where no co-payment has been charged to the patient and the provider claims reimbursement for the service directly from Medicare, Australia's universal health insurance scheme. There were no areas in regional areas that were in the 4th quartile for bulk-billing. In addition, there were no areas in outer regional areas that were in the 1st quartile for OPC.

Supplementary Table S6. Association of PC characteristics with long consultations, odds ratio and 95% confidence interval, by region

	GP FTE OR (95%CI)	***Out-of-pocket costs*** OR (95%CI)	***Bulk-billing*** OR (95%CI)	***After-hours care*** OR (95%CI)
**Cities**				
1st quartile (ref)	1	1	1	1
2nd quartile	1.07 (0.85–1.36)	1.04 (0.89–1.22)	1.03 (0.83–1.27)	0.95 (0.62–1.46)
3rd quartile	0.99 (0.78–1.26)	1.20 (0.98–1.46)	1.00 (0.83–1.21)	0.90 (0.59–1.37)
4th quartile	1.27 (1.00–1.60)	1.16 (0.99–1.37)	0.87 (0.73–1.03)	0.84 (0.55–1.28)
p-value for term	**0.007**	0.165	0.14	0.449
ICC	0.01	0.011	0.011	0.012
PCV (%)	27	14	14	9
MOR	1.19	1.2	1.2	1.21
**Inner regional**				
1st quartile (ref)	1	1	1	1
2nd quartile	1.25 (0.89–1.75)	1.46 (0.92–2.31)	1.47 (1.12–1.91)	1.37 (1.07–1.76)
3rd quartile	0.98 (0.7–1.37)	1.08 (0.69–1.70)	1.38 (1.05–1.82)	1.39 (0.90–2.13)
4th quartile	1.32 (0.97–1.80)	0.98 (0.61–1.57)	–	1.48 (1.07–2.06)
p-value for term	0.218	**0.029**	**0.011**	**0.024**
ICC	0.027	0.024	0.024	0.024
PCV (%)	13	23	22	24
MOR	1.33	1.31	1.31	1.31
**Outer regional**				
1st quartile (ref)	1	–	1	–
2nd quartile	0.93 (0.63–1.38)	ref	1.17 (0.88–1.57)	–
3rd quartile	0.94 (0.61–1.47)	0.64 (0.48–0.85)	1.57 (1.20–2.05)	–
4th quartile	0.67 (0.43–1.04)	0.56 (0.42–0.73)	–	–
p-value for term	0.362	**<.001**	**0.004**	**–**
ICC	0.036	0.022	0.022	–
PCV (%)	12	47	46	–
MOR	1.4	1.29	1.3	–

Notes: PC, primary care; OR, odds ratio; CI, confidence interval; GP, general practitioner; FTE, full-time equivalent; OPC, out-of-pocket costs; ICC, Intra-class coefficients; PCV, proportional change in variance; MOR, median odds ratio. Odds ratio and 95% confidence intervals shown, Wald joint test of significance shown. Significant terms bolded. 1st quartile corresponds to 25% of the population in the lowest category, 4th quartile 25% of the population in the highest category. Models adjusted for individual sociodemographic and need variables. PCV reported change from model adjusted for sociodemographic and need variables (without area-level variable). Bulk-billing refers to where no co-payment has been charged to the patient and the provider claims reimbursement for the service directly from Medicare, Australia's universal health insurance scheme. There were no areas in regional areas that were in the 4th quartile for bulk-billing. In addition, there were no areas in outer regional areas that were in the 1st quartile for OPC.

.

Supplementary Table S7. Association of PC characteristics with care planning, odds ratio and 95% confidence interval, by region

	GP FTE	*Out-of-pocket costs*	*Bulk-billing*	*After-hours care*
	**OR (95%CI)**	**OR (95%CI)**	**OR (95%CI)**	**OR (95%CI)**
**Cities**				
1st quartile (ref.)	1	1	1	1
2nd quartile	0.74 (0.47–1.16)	0.86 (0.70–1.06)	1.52 (1.16–2.01)	2.32 (1.16–4.66)
3rd quartile	0.77 (0.49–1.21)	0.85 (0.65–1.12)	1.90 (1.50–2.42)	3.00 (1.50–5.96)
4th quartile	0.67 (0.43–1.06)	0.51 (0.41–0.64)	2.12 (1.69–2.66)	3.06 (1.54–6.09)
p-value for term	0.375	**<.001**	**<0.001**	**0.003**
ICC	0.036	0.02	0.018	0.029
PCV (%)	7	48	54	26
MOR	1.4	1.28	1.26	1.35
**Inner regional**				
	1	1	1	1
2nd quartile	1.08 (0.70–1.66)	0.84 (0.46–1.55)	1.74 (1.27–2.38)	0.84 (0.59–1.18)
3rd quartile	0.97 (0.63–1.49)	0.88 (0.48–1.60)	1.50 (1.08–2.07)	1.26 (0.70–2.27)
4th quartile	1.45 (0.98–2.15)	0.59 (0.31–1.10)	–	0.91 (0.57–1.43)
p-value for term	0.274	0.132	**0.002**	0.526
ICC	0.042	0.04	0.033	0.044
PCV (%)	12	17	31	8
MOR	1.44	1.42	1.37	1.45
**Outer regional**				
	1	–	1	–
2nd quartile	0.95 (0.63–1.45)	1	1.27 (0.90–1.81)	–
3rd quartile	0.86 (0.54–1.38)	0.74 (0.52–1.05)	1.66 (1.20–2.30)	–
4th quartile	0.67 (0.42–1.07)	0.62 (0.44–0.87)	–	–
p-value for term	0.402	**0.022**	**0.009**	**–**
ICC	0.039	0.032	0.031	–
PCV (%)	12	28	30	–
MOR	1.42	1.37	1.36	–

Notes: PC, primary care; OR, odds ratio; CI, confidence interval; GP, general practitioner; FTE, full-time equivalent; OPC, out-of-pocket costs; ICC, Intra-class coefficients; PCV, proportional change in variance; MOR, median odds ratio. Odds ratio and 95% confidence intervals shown, Wald joint test of significance shown. Significant terms bolded. 1st quartile corresponds to 25% of the population in the lowest category, 4th quartile 25% of the population in the highest category. Models adjusted for individual sociodemographic and need variables. PCV reported change from model adjusted for sociodemographic and need variables (without area-level variable). Bulk-billing refers to where no co-payment has been charged to the patient and the provider claims reimbursement for the service directly from Medicare, Australia's universal health insurance scheme. There were no areas in regional areas that were in the 4th quartile for bulk-billing. In addition, there were no areas in outer regional areas that were in the 1st quartile for OPC.

Supplementary Table S8. Cross-level effect modification for care planning: Odds ratio and 95% confidence interval for area PC service characteristics (continuous) and as interaction with education, separately by region.

PHC characteristic and interaction terms	Cities<br>OR (95%CI)	Inner regional<br>OR (95%CI)	Outer regional/ remote<br>OR (95%CI)
GP FTE			
No school certificateXGP FTE	1	–	–
School certificateXGP FTE	0.949 (0.630–1.428)	–	–
Apprentice/diplomaXGP FTE	1.080 (0.713–1.637)	–	–
UniversityXGP FTE	0.923 (0.609–1.398)	–	–
P-value	0.777	–	–
OPC			
No school certificateXOPC	1	–	1
School certificateXOPC	1.001 (0.981–1.022)	–	1.008 (0.972–1.046)
Apprentice/diplomaXOPC	0.995 (0.974–1.015)	–	0.981 (0.944–1.018)
UniversityXOPC	0.999 (0.978–1.020)	–	1.012 (0.964–1.062)
P-value	0.824	–	0.31
Bulk-billing			
No school certificateXbulk-billing	1	1	1
School certificateXbulk-billing	1.001 (0.994–1.008)	1.006 (0.997–1.014)	1.000 (0.992–1.009)
Apprentice/diplomaXbulk-billing	1.003 (0.997–1.010)	0.999 (0.991–1.008)	1.007 (0.998–1.017)
UniversityXbulk-billing	1.002 (0.995–1.010)	0.996 (0.986–1.006)	0.999 (0.987–1.010)
P-value	0.66	0.1	0.18
After-hours care			
No school certificateXafter-hours	1	–	–
School certificateXafter-hours	0.998 (0.974–1.022)	–	–
Apprentice/diplomaXafter-hours	0.992 (0.967–1.017)	–	–
UniversityXafter-hours	1.007 (0.980–1.035)	–	–
P-value	0.605	–	–

Notes: Models additionally adjusted for individual age, sex, marital status, country of birth and need variables (self-reported health, number of chronic conditions, physical functioning). Continuous variable and interaction term with education for each area PHC characteristic added separately. P-value, Wald test of joint significance for addition of cross-level interaction term. Significant terms bolded. Terms not reported did not have a monotonic relationship as a categorical variable with outcome. After-hours care not tested in outer regional/ remote areas due to unreliability of this estimate in this region. CD Care not tested with long consultations given uncertain interpretation. Abbrev. PC, primary care; OR, odds ratio; CI, confidence interval; FTE, full-time equivalent; GP, general practitioner; OPC, out-of-pocket cost. Bulk-billing refers to where no co-payment has been charged to the patient and the provider claims reimbursement for the service directly from Medicare, Australia's universal health insurance scheme.
